# Injurious Administration of the Sulphate of Quinine

**Published:** 1833

**Authors:** 


Art. II. *Injurious administration of the Sulphate of Quinine.*—In the Journal just quoted, our friend Dr. Monett, of Washington, Mississippi, has published an interesting paper on the uses and abuses of this medicine, which he ranks with digitalis and tartarized antimony, denying to it a tonic power. We have more than once in *this* Journal, asserted its claims to the character of a narcotic sedative, and denied to it the character which belongs to the Bark, from which it is prepared. The following extract may be read with advantage by those who regard it as a tonic and stimulant.

"The *bark* having been used in all cases of remittent and intermittent fevers indiscriminately, must necessarily have failed to cure those cases in which its use was improper. The same result will obtain from a similar use of quinine. The *bark is improper and pernicious* in all cases characterized by general increased arterial tone and action; or in fevers consequent upon local inflammation or engorgement, and where the tone of the system is unimpaired. If administered in such cases it will certainly disappoint expectation. The best Peruvian bark possesses a tonic and a febrifuge property combined. This combination of properties adapts it peculiarly to cases of fevers and febrile affections based upon an *asthenic diathesis.* This circumstance, in all cases, should form the criterion for its use, whatever be the name of the disease or its external character. The same property which adapts the bark to this class of diseases, renders it in the same degree pernicious in those of an opposite character.

The *quinine* is salutary and proper, as a remedial agent, in cases the reverse of those in which the bark is applicable. The quinine, which has been erroneously supposed to contain the whole tonic property of the bark, is in fact its *febrifuge* essence uncombined and *without any tonic property whatever.* Thus, when quinine is improperly given, its pernicious effects are much more aggravated than those resulting from an improper use of the bark. For in the latter the febrifuge in some degree counteracts the tonic property, and *vice versa:* besides, the improper cases for bark, are those states in which the system is unimpaired by debility, and can withstand a temporary error without serious injury. On the other hand, the quinine exerts its febrifuge powers *by directly diminishing the force* and tone of the arterial system, and is injurious only in those cases where the asthenic diathesis prevails. In such cases, with an impaired state of the constitutional powers, atony in the circulating system, and all the train of rapid and fatal symptoms attendant upon adynamic fevers, even a temporary diminution of the powers of life cannot be sustained without the most imminent hazard. When used in these cases, quinine, unlike digitalis, tart. ant., &c., instead of diminishing the force and frequency of the arterial pulsations, diminishes their force, but *increases their frequency,* together with all the signs of irritable debility. This is what M. Bally means by its irritating property; and this is what others have *erroneously considered its stimulant effects.* For while quinine acts as a febrifuge and contra-stimulant in sthenic diseases, it produces the highest grade of irritable excitement in those of direct debility.

But in cases of open excitement, in vigorous constitutions, the quinine may be used advantageously in most of those cases of autumnal remittents and intermittents, in which tartarized antimony, digitalis, pulv. ipecac. c., saline diaphoretics, and medicines of that class might be safely employed. In such cases the system will not unfrequently bear large doses of quinine without any visible effect; as is likewise often the case with tart. ant. and other medicines. *But it cannot be otherwise than pernicious in all asthenic cases;* and I consider highly preposterous an attempt to make it supercede many of our valuable remedies in the treatment of summer and autumnal fevers, especially in this climate and latitude, where most of our summer and autumnal diseases originate in the debilitating and relaxing effects of heat.

It is truly a matter of astonishment, that this article should have been forced so extensively into the southern practice, where diseases, especially summer and autumnal fevers, are least adapted to its use. Yet such is the force of habit and preconceived notions in medicine, as in every thing else, that too often they usurp the province of judgment and discrimination; and medicines are often administered in certain cases, only because we have been in the habit of prescribing them in others. And thus it is with calomel and quinine. I doubt whether mercury itself, in so short a period, has ever produced more pernicious effects than quinine. The effects of the former are known and visible to all; it is therefore given with caution. The latter, under the misguided notion of its tonic and stimulant properties, is administered in the most improper cases; while all its pernicious effects are reckoned only so many symptoms of a disease, which it is most unquestionably to cure. The more alarming these symptoms are, the more the pernicious article is pressed upon the patient, aggravating every alarming symptom, until the unfortunate victim is pressed into the grave; the price of being cured *secundum artem* by quinine! I have often been called to see patients whose case was considered hopeless, where the disease was protracted into that state solely by the use of quinine, and where the only important indication towards effecting a cure was the discontinuance of *the medicine.* I have seen physicians use the sul. quinæ, without any fixed rule, or any definite views relative to its *modus operandi,* in all cases and in all stages and varieties of fever indiscriminately, like a charm or enchanted dose, which is to adapt itself to every peculiarity of case, and to effect wonders they know not how. Even in this manner it cannot always fail. Some cases will happen to be proper, and the effects favorable. This will cause it to be given again in twenty improper cases. So egregiously are its pernicious effects overlooked or misconceived, that not unfrequently the most decided evidence of its pernicious effects, such as tinnitus aurium, deafness, vertigo, strabismus, cephalalgia, nausea, and vomiting, are construed into criteria for its administration, and as indicative of its salutary effects. Effects these, which I do not hesitate to denounce, as certain indications of its improper use, either as relates to the case, or the quantity administered.

*The sulphate of quinine,* I repeat, *is a valuable febrifuge and counter-stimulant, and is not in any sense a tonic.* Yet its febrifuge effects may be obtained without injury from its counter-stimulant properties, even in slight cases of asthenic fever, or its action may be so modified by a judicious combination of aromatic stimulants and opiates, that in cases of moderate asthenia, no detriment will be sustained from its contra-stimulant properties. In this respect it bears a strong reverse analogy with camphor. The quinine being contra-stimulant, requires its action to be guarded by capsicum, camphor, and opium. Camphor, on the other hand, being stimulant, requires the addition of antimonials and other contra-stimulant diaphoretics, to adapt it to cases in which it would otherwise be inadmissible. However, *quinine should not be*

*used in any manner in simple debility unattended with fever.* The danger lies in administering quinine in cases attended with a cool, relaxed skin, and general diminution of tone and action. There must be febrile action, or quinine is injurious under any combination.

It may be laid down as a general rule, to which there will be but few exceptions, that the sul. quinæ, as a remedy, is admissible only in persons of strong athletic habits, of sanguineous or bilious temperaments, and whose systems are not debilitated by previous protracted disease,and in those who are temperate and free from any constitutional irritability. Diseases supervening upon such constitutions and habits, will be mostly those of direct excitement, and which may in some stage be treated with quinine. The quinine, however, is contra-indicated in all individuals of lax fibre, and feeble circulation; in leucophlegmatic and nervous temperaments,* attended with torpor in the absorbent system, and with adipose depositions. In these constitutions and habits, diseases usually in this latitude assume the asthenic character, especially summer and autumnal fevers, and constitutional derangements consequent upon them.

It may not be superfluous to be more particular in specifying those diseases in which *the sulphate of quinine is pernicious.* They are chronic debility from whatever cause; want of tone in the ultimate fibre, with defective energy, mental and corporeal; disorders consecutive of weak or irregular, defective or increased action in the abdominal viscera. The external evidences of such cases are pale skin, sallow complexion; feeble and quick pulse, or weak and sluggish; pale or ash-coloured tongue, not furred; clear white sclerotica; mental lethargy, and aversion to bodily exertion; irregular biliary discharges, sometimes deficient, often profuse, thin and yellow: in extreme cases of irritable debility, there is a tendency to syncope in a sitting or erect posture, throbbing in the head, tinnitus aurium, palpitation and vertigo. Quinine aggravates every one of these symptoms. It is improper in violent congestive fever, with cerebral congestion, and in the collapsed stage of common remittent, malignant, and typhus fevers. In any and all cases of these, quinine, as it is generally administered, *never fails even in small doses, to induce a quicker and more irritable pulse, thirst, and cerebral congestion,* and a train of unfavorable symptoms. These, which are aggravated by each dose of quinine, are readily relieved by wine or toddy, aromatic cordials, camphor, ammonia, and opiates.

Another disease, whose course has been most terrific in our own country, as well as elsewhere, is rapidly hurried on in its fatal termination by quinine. Those who have contracted a partiality for this article, have naturally enough conceived it peculiarly adapted to the cure of *malignant cholera,* (cholera asphyxia.) The *cholera* has uniformly exhibited the character and symptoms of a most malignant collapsed fever, and has always, in my opinion, required a liberal exhibition of

*M. Bland, in the summer of 1825, observed deafness and other symptoms of cerebral congestion, &c., produced by large doses of quinine. This he observed chiefly in persons of *tall and slender make, and of nervous temperaments.*—*Am. Journ. Med. Sciences, No. II.*

aromatic stimulants, camphor, æther, and calomel. The most successful have been calomel, opium, camphor, and capsicum. If these articles be beneficial, in their operation, and if quinine be a contra-stimulus, as it undoubtedly is, the impropriety of its use in this disease, however guarded by other combinations, is evident.

In the common *congestive* summer and autumnal fevers of this country, we are presented with an imperfect circulation; abdominal and thoracic congestions; pale, relaxed, and cold skin, sometimes covered with profuse cold sweat; pulse small, and almost imperceptible; cold extremities; impaired sensibility, and a general derangement of all the secernant functions. The vital energies are defective, or are at once depressed by the force of disease below the point of reaction; the whole system is, in fact, in a stage of collapse, from which nothing can rouse it, but the prompt use of the most powerful stimuli internally and externally. It is in fevers of this character, that quinine is too often freely used, under the erroneous impression that it is a stimulant and tonic. It only makes the fatal catastrophe more certain. If quinine, under any circumstance, be capable of removing congestion, it is only in vigorous constitutions, where the circulation is only partially oppressed, and where the external heat is not reduced below a normal state.

There are other cases likewise, in which quinine is pernicious. One is that peculiarly irritable and debilitated state of the whole animal functions most common in females, designated by the term *anemia,* (parops hæmatosis.) In cases of this kind, where corroborants and generous diet are essentially necessary, the judicious physician cannot administer quinine without speedily perceiving its pernicious tendency. In *gangrene* the sul. quinæ is highly pernicious. I should not have supposed it necessary to name this as an improper case for quinine, had I not seen it recommended as a substitute for bark and wine in gangrenous inflammation in a most respectable work on surgery.* I do decidedly believe there is no disease in the whole range of nosology, in which the use of this article would be more pernicious than in extensive gangrenous inflammation.

* Principles of Surgery. By James Syme, F. R. S. E. &c.

